# Reviewer acknowledgement

**DOI:** 10.1186/s13049-015-0100-8

**Published:** 2015-02-26

**Authors:** Hans Morten Lossius, Kjetil Søreide, Kristi G Bache

**Affiliations:** Norwegian Air Ambulance Foundation, Drøbak, Norway; Stavanger University Hospital, Stavanger, Norway

## Abstract

The *Scandinavian Journal of Trauma, Resuscitation and Emergency Medicine* is extremely grateful for the time, hard work and support of its highly-qualified peer reviewers. The editors of the *Scandinavian Journal of Trauma, Resuscitation and Emergency Medicine,* the Norwegian Air Ambulance Foundation and BioMed Central would like to show our appreciation by thanking the following people for their assistance reviewing manuscripts for the journal in 2014.

**Fikri Abu-Zidan**

United Arab Emirates

**Hemant Agarwal**

United States of America

**Vanessa Agostini**

Italy

**Ali Akar**

Turkey

**Jennifer Albrecht**

United States of America

**Gidon Almogy**

Israel

**Badr Alsayed**

Saudi Arabia

**Kurt Ammer**

Austria

**Hagen Andruszkow**

Germany

**Asa Axelsson**

Sweden

**Miklosh Bala**

Israel

**Zsolt Balogh**

Australia

**Graham Barker**

United Kingdom

**David Bar-Or**

United States of America

**Kristian Bartnes**

Norway

**Ceri Battle**

United Kingdom

**Thomas Bein**

Germany

**Fabio Beltrame**

Italy

**Peleg Ben-Galim**

Israel

**William Berger**

Brazil

**Sebastian Bergrath**

Germany

**Joost Bierens**

Netherlands

**Conrad Arnfinn Bjørshol**

Norway

**Xavier Bobbia**

France

**Christa Boer**

Netherlands

**Viktoria Bogner**

Germany

**Massimo Bonacchi**

Italy

**Andrea Borghini**

Italy

**Omar Bouamra**

United Kingdom

**Mal Boyle**

Australia

**John Bro-Jeppesen**

Denmark

**Gerard Bury**

Ireland

**Michael Busch**

Norway

**Daniel Candinas**

Switzerland

**Tom Carrell**

United Kingdom

**Enrique Casalino**

France

**Maaret Castrén**

United States of America

**Chia-Ter Chao**

Taiwan

**Ray-Jade Chen**

Taiwan

**Ivy Cheng**

Canada

**Ian Civil**

New Zealand

**Tim Coats**

United Kingdom

**Stephen Cohn**

United States of America

**G Colville**

United Kingdom

**Roberto Copetti**

Italy

**Charles Court-Brown**

United Kingdom

**Kate Crewdson**

United Kingdom

**Nicola Curry**

United Kingdom

**Jeremy Dale**

United Kingdom

**Jan De Waele**

Belgium

**Matthias Derwall**

Germany

**Salomone Di Saverio**

Italy

**Ahmadreza Djalali**

Italy

**Nurettin Ozgur Dogan**

Turkey

**David Dries**

United States of America

**Trygve Eftestøl**

Peru

**Sharon Einav**

Israel

**Ulf Ekelund**

Sweden

**Mazen El Sayed**

Lebanon

**Kashif Faiz**

Norway

**Sabina Fattah**

Norway

**Marc Felzen**

Germany

**Karim Fikry**

United States of America

**Matthias Fischer**

Germany

**Bernhard Floerchinger**

Germany

**Hans Friberg**

Sweden

**Ari Friedman**

United States of America

**Erika Frischknecht Christensen**

Denmark

**Daniel Frith**

United Kingdom

**Margaret Fry**

Australia

**Peter Fu**

Taiwan

**Belinda Gabbe**

Australia

**Samuel Galvagno**

United States of America

**Jeff Garner**

United Kingdom

**Margrete Gaski**

Norway

**Tomasz Gaszynski**

Poland

**Leo Geeraedts**

Netherlands

**Mikael Gellerfors**

Sweden

**Poya Ghorbani**

Sweden

**Sven Erik Gisvold**

Norway

**Ernestina Gomes**

Portugal

**Zsigmond Göndöcs**

Hungary

**Wolfgang Grechenig**

Austria

**David Griffiths**

United Kingdom

**Nils Haake**

Germany

**Carl Haasper**

Germany

**Jostein S Hagemo**

Norway

**Peter Hallas**

Denmark

**Kiran Hebbar**

United States of America

**Catherine Heim**

Switzerland

**Petr Hejna**

Czech Republic

**Eirik Helseth**

Norway

**Jon-kenneth Heltne**

Norway

**Robin Hemphill**

United States of America

**Bard Heradstveit**

Norway

**Johan Herlitz**

Sweden

**Manuel E. Herrera Gutierrez**

Spain

**Rasmus Hesselfeldt**

Denmark

**Martin Hoenigl**

Austria

**Maren Ranhoff Hov**

Norway

**Stefan Huber-Wagner**

Germany

**Steinar Hunskaar**

Norway

**Per Kristian Hyldmo**

Norway

**Matthias Jaeger**

Australia

**Jan Jansen**

United Kingdom

**Helena Jjäntti**

Finland

**Per Ingemar Johansson**

Denmark

**Alan Wayne Jones**

Sweden

**Iosifina Karmiolou**

Greece

**Jeffry Kashuk**

United States of America

**Christina Katsios**

Canada

**Fulvio Kette**

Italy

**Salma Khan**

Pakistan

**Tareq Kheirbek**

United States of America

**Kosuke Kiyohara**

Japan

**Andreas J. Kruger**

Norway

**Jouni Kurola**

Finland

**Martin Kurz**

Norway

**Alf Inge Larsen**

Norway

**Peter Larsen**

New Zealand

**Annmarie Lassen**

Denmark

**Rifat Latifi**

United States of America

**Zeljko Lausevic**

Serbia

**Luke Leenen**

Netherlands

**Rolf Lefering**

Germany

**Mark Lehnert**

Germany

**Lisa Leon**

United States of America

**Lai Yee Leung**

United States of America

**Liu-Dong Li**

China

**Christian Lindskov**

Denmark

**Grant Lipman**

United States of America

**Anne Lippert**

Denmark

**David Lockey**

United Kingdom

**Kristin Long**

United States of America

**Kare Lovstakken**

Australia

**Adam Lund**

Canada

**Richard Lyon**

United Kingdom

**Jan Erik Madsen**

Norway

**Tracy Madsen**

United States of America

**Marc Maegele**

Germany

**Carolina Malta Hansen**

Denmark

**Meghan Marsac**

United States of America

**Aidan Marsh**

United Kingdom

**Blanca Martinez**

Italy

**Antonio Massarutto**

Italy

**Walter Mauritz**

Austria

**Lauralyn McIntyre**

Canada

**Jane McVicar**

United Kingdom

**Reitske Meganck**

Belgium

**Terje Meling**

Norway

**Christine Migliorini**

Australia

**Timothy Miller**

United States of America

**Taro Minami**

United States of America

**Robert Minns**

United Kingdom

**Oscar Miro**

Spain

**Alessandro Morandi**

Italy

**Andrea Morelli**

Italy

**Umberto Morelli**

France

**Nicolas Mpotos**

Belgium

**Pål Aksel Næss**

Norway

**Thach Nguyen**

United States of America

**Vicki Noble**

United States of America

**Jerry Nolan**

United Kingdom

**Trond Nordseth**

Norway

**Yutaka Oda**

Japan

**Mikael Öman**

Sweden

**Per Örtenwall**

Sweden

**Peter Paal**

Austria

**Kyrre Pedersen**

Sweden

**Kobi Peleg**

Israel

**Carmen Andrea Pfortmueller**

Switzerland

**Ornella Piazza**

Italy

**Johan Pillgram-Larsen**

Norway

**Søren Erik Pischke**

Norway

**Konstanze Plaschke**

Germany

**Alison Porter**

United Kingdom

**Rahul Raj**

Finland

**Catarina Raposo**

Brazil

**Stephen Rashford**

Australia

**Kristina Rehm**

United States of America

**Marius Rehn**

Norway

**Zaccaria Ricci**

Italy

**Louis Riddez**

Sweden

**Rune Rimstad**

Norway

**Jukka Ristiniemi**

Finland

**Antonio Rodríguez Núñez**

Spain

**Leif Rognas**

Denmark

**Sten Rubertsson**

Sweden

**Samy Sadek**

United Kingdom

**Joshua Salzman**

United States of America

**Simone Savastano**

Italy

**Thomas Scalea**

United States of America

**Herbert Schoumlchl**

Austria

**Juergen Schaefer**

Germany

**Thomas Andersen Schmidt**

Denmark

**Nicolai A. Schultz**

Denmark

**Christopher Seymour**

United States of America

**Sang Do Shin**

Republic of Korea

**Katrin Singler**

Germany

**Simon Skibsted**

United States of America

**Markus Skrifvars**

Finland

**Kerstin Sluys Prignitz**

Sweden

**Karl Heinz Smolle**

Austria

**Curtis P Snook**

United States of America

**Stephen J M Sollid**

Norway

**Eldar Søreide**

Norway

**Andreas Sorlie**

Norway

**Konstantinos Spaniolas**

United States of America

**Philp Spinella**

United States of America

**Philip Stahel**

United States of America

**Petter Andreas Steen**

Norway

**Jacob Steinmetz**

Denmark

**Harald Steinum**

Norway

**Christian Storm**

Germany

**Anneli Strömsöe**

Sweden

**Geir Arne Sunde**

Norway

**Terje Sundstrøm**

Norway

**Hildigunnur Svavarsdottir**

Iceland

**Frank Tacke**

Germany

**Korhan Taviloglu**

Turkey

**Brian Telesz**

United States of America

**Oyvind Thomassen**

Norway

**Kenneth Thorsen**

Norway

**Else Tonnesen**

Denmark

**Oddvar Uleberg**

Norway

**Javier Urbano**

Spain

**Caesar Ursic**

United States of America

**Fabrice Vallee**

France

**Esther Van Lieshout**

Netherlands

**Morten Vetrhus**

Vetrhus

**François Vincent**

France

**Wolfgang Voelckel**

Austria

**Jyrki Vuola**

Finland

**Charles Wade**

United States of America

**Jon Walker**

United Kingdom

**Beat Walpoth**

Switzerland

**Hao Wang**

United States of America

**Andrew Weatherall**

Australia

**Guy Weinberg**

United States of America

**Bridget Wells**

United Kingdom

**Richard Williams**

United Kingdom

**Des Winter**

Ireland

**Torben Wisborg**

Norway

**Cherylynn Wium**

South Africa

**Marzena Wojewódzka-Zelezniakowicz**

Poland

**Richard Wolfe**

United States of America

**Thomas Wurmb**

Germany

**Lucia Zannini**

Italy

